# Genetic correction of haemoglobin E in an immortalised haemoglobin E/beta-thalassaemia cell line using the CRISPR/Cas9 system

**DOI:** 10.1038/s41598-022-19934-7

**Published:** 2022-09-16

**Authors:** Kongtana Trakarnsanga, Nontaphat Thongsin, Chanatip Metheetrairut, Chartsiam Tipgomut, Saiphon Poldee, Methichit Wattanapanitch

**Affiliations:** 1 Department of Biochemistry, Faculty of Medicine Siriraj Hospital, Mahidol University, Bangkok, Thailand; 2 Research Department, Faculty of Medicine Siriraj Hospital, Siriraj Center for Regenerative Medicine, Mahidol University, Bangkok, Thailand; 3 Department of Immunology, Faculty of Medicine Siriraj Hospital, Mahidol University, Bangkok, Thailand

**Keywords:** Haematopoiesis, Haematological diseases, Gene therapy

## Abstract

β-thalassaemia is one of the most common genetic blood diseases worldwide with over 300 mutations in the HBB gene affecting red blood cell functions. Recently, advances in genome editing technology have provided a powerful tool for precise genetic correction. Generation of patient-derived induced pluripotent stem cells (iPSCs) followed by genetic correction of HBB mutations and differentiation into haematopoietic stem/progenitor cells (HSPCs) offers a potential therapy to cure the disease. However, the biggest challenge is to generate functional HSPCs that are capable of self-renewal and transplantable. In addition, functional analyses of iPSC-derived erythroid cells are hampered by poor erythroid expansion and incomplete erythroid differentiation. Previously, we generated an immortalised erythroid cell line (SiBBE) with unique properties, including unlimited expansion and the ability to differentiate into mature erythrocytes. In this study, we report a highly efficient genetic correction of HbE mutation in the SiBBE cells using the CRISPR/Cas9 system. The HbE-corrected clones restored β-globin production with reduced levels of HbE upon erythroid differentiation. Our approach provides a sustainable supply of corrected erythroid cells and represents a valuable model for validating the therapeutic efficacy of gene editing systems.

## Introduction

β-thalassaemia is one of the most common monogenic diseases with defects in the synthesis of β-globin, a component of adult haemoglobin, which is responsible for the oxygen delivery function of red blood cells. Over 300 mutations in and around the beta-globin gene have been identified as causes of β-thalassaemia^1^. HbE/β-thalassaemia, a severe disease, affects over a million people worldwide, particularly in Asia^2^. It is caused by compound heterozygous mutations in which a mutation of one allele causes reduction or absence of β-globin production depending on the type of mutation and a mutation of the other allele that causes haemoglobin E^3^. Haemoglobin E is caused by a point mutation at codon 26 of the β-globin gene, GAG → AAG, resulting in an amino acid substitution (from glutamic acid to lysine), which reduces the binding ability between α- and β-globins. This mutation also acts as an alternative splice site which decreases the amount of messenger RNA and further decreases β-globin synthesis^4^. The abnormal β-globin chain synthesis in HbE/β-thalassaemia contributes to globin chain imbalance, ineffective erythropoiesis, oxidative damage, apoptosis and shortened red cell survival^2^. The most common manifestations are similar to those of other severe β-thalassaemia, including anaemia, jaundice, hepatosplenomegaly, growth retardation, and thalassaemic facies^4^. At present, a standard treatment for most thalassaemia patients is regular blood transfusions, with side effects such as iron overload and allo-immunisation. The only curative treatment is bone marrow transplantation, which is limited by the availability of human leukocyte antigen (HLA)-matched stem cell donors^5,6^.

Induced pluripotent stem cells (iPSCs) offer a renewable source of patient- or disease-specific cells for disease modelling, including genetic and acquired haematological disorders^7^. In the past decades, several studies reported the genetic correction of beta-thalassaemia in patient-derived iPSCs using different gene editing technologies such as zinc-finger nucleases (ZFNs), transcription activator-like effector nuclease (TALENs) and CRISPR/Cas9 systems^8–15^. Although these studies successfully edited the genetic mutations in the beta-globin (*HBB*) gene and induced the edited iPSCs into erythroblasts, the cells exhibited very low levels of adult globin protein compared to those in adult red blood cells. In addition, these erythroblasts had a very low proliferation rate and enucleation efficiency, making them unsuitable for drug screening or developing therapeutic approaches to genetically correct the mutations^16^.

Recently, we reported the establishment of the immortalised erythroid cell line, Siriraj Bristol Beta-thalassaemia/haemoglobin E cell line; SiBBE, from haematopoietic stem cells (HSCs) of a patient with HbE/beta-thalassaemia^17^. Notably, erythroblasts differentiated from the SiBBE cells exhibited the haemoglobin profile recapitulating those of the patient’s red blood cells and could enucleate to generate mature reticulocytes. Therefore, the SiBBE cell line represents a renewable source of erythroblasts suitable for studying underlying disease pathology, drug screening as well as developing a therapeutic strategy using genome editing technology.

In this study, we performed genetic correction of HbE mutation in the SiBBE cell line using the CRISPR/Cas9 ribonucleoprotein (RNP) complex and the modified single-stranded oligonucleotide (ssODN) donor template. This approach could efficiently correct the HbE mutation and generate the HbE-corrected SiBBE subclones with the ability to differentiate into mature erythrocytes. Unlike the HbE-corrected iPSC-derived erythroid cells, which mainly produced foetal haemoglobin, these HbE-corrected erythrocytes restored the production of mature beta haemoglobin compared to the untransfected control. Our study demonstrated the usefulness of the immortalised erythroid cell line as a study model for genetic correction and offered a renewable source of red blood cells for cell-based therapy in thalassaemia.

## Results

### Correction of HbE mutation in SiBBE cells using the CRISPR/Cas9 system

**Figure 1:**
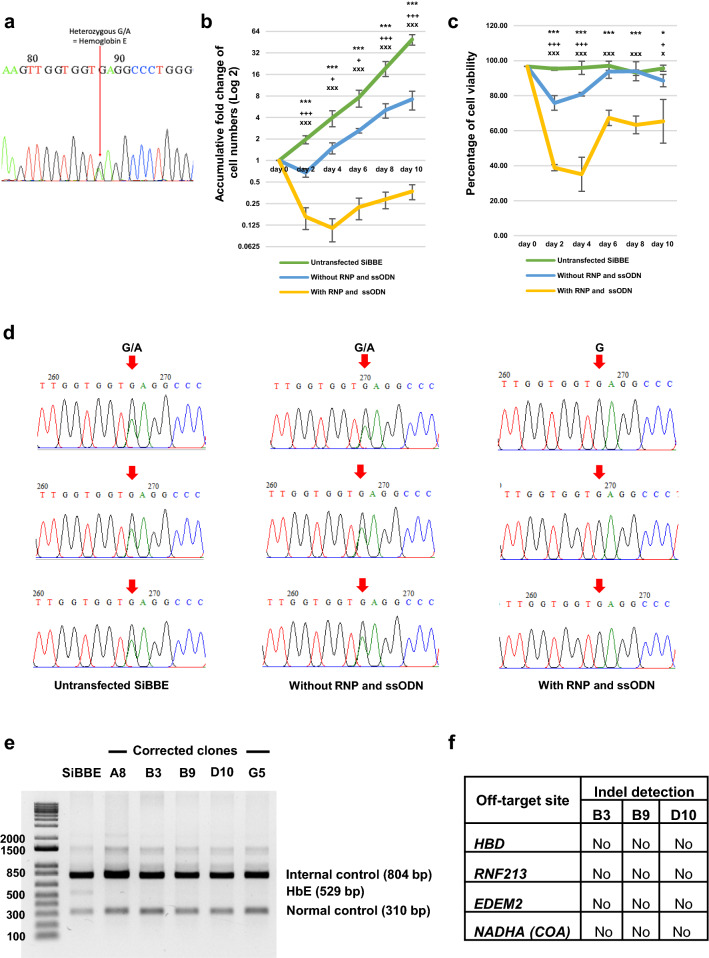
Correction of HbE mutation in the SiBBE cell line using the CRISPR/Cas9 system. (**a**) Beta globin gene was PCR amplified from genomic DNA and sequenced by standard Sanger sequencing. Heterozygous allele at *HBB*: c.79G > A in exon 1, creating haemoglobin E (HbE) allele. (**b**) Accumulative fold expansion of the nucleofected SiBBE cells with and without the RNP and 100 pmol of the ssODN donor template. The untransfected SiBBE cells were used as a control (n = 3). (**c**) Viability of the nucleofected SiBBE cells with and without the RNP and 100 pmol of the ssODN donor template. The untransfected SiBBE cells were used as a control (n = 3). For both (**b**) and (**c**), * *p* value < 0.05, *** *p* value < 0.01 (between the nucleofected SiBBE cells with the RNP and ssODN and the untransfected SiBBE cells); + *p* value < 0.05, +++ *p* value < 0.01 (between the nucleofected SiBBE cells without the RNP and ssODN and the untransfected SiBBE cells); x *p* value < 0.05, xxx *p* value < 0.01 (between the nucleofected SiBBE cells with and without the RNP and ssODN), two-tailed t-test. (**d**) Beta-globin gene was PCR amplified from genomic DNA and sequenced by standard Sanger sequencing using the forward primer. HbE mutation, a heterozygous G/A mutation as indicated by red arrows, was identified in the untransfected SiBBE cells and the nucleofected SIBBE cells without the RNP and ssODN but was not detected in the nucleofected SiBBE cells with the RNP and ssODN. (**e**) Multiplex PCR analysis for HbE mutation in five subclones compared to the parental SiBBE cells. A product size of 529 bp represents the HbE point mutation. (**f**) A summary of Sanger sequencing results of potential off-target sites for sgRNA as identified by BLAST search. Three subclones, B3, B9 and D10, were chosen as representatives.

Prior to the genetic correction of HbE mutation in the SiBBE cells, the expanding SiBBE cells (day 62) were harvested for DNA extraction, amplification, and sequencing to confirm the existence of HbE mutation in the cell line (Fig. 1a). To correct the HbE mutation, we nucleofected the SiBBE cells with the ribonucleoprotein complex (RNP) and 100 pmol of the ssODN donor template. The SiBBE cells were also nucleofected without the RNP and ssODN donor template as a control sample. On day 2 after transfection, a decrease in cell number was observed in the transfected cells without the RNP and ssODN while a drastic decrease in cell number was observed in the nucleofected cells with the RNP and ssODN and was carried on to day 4 with a total of approximately ninefold decrease (Fig. 1b). The numbers of the nucleofected cells with and without the RNP and ssODN were significantly lower than those of the untransfected cells throughout the culture (n = 3) (Fig. 1b). Moreover, the numbers of the nucleofected cells with the RNP and ssODN were significantly lower than those of the nucleofected cells without RNP and ssODN throughout the culture (n = 3) (Fig. 1b). On day 2 after nucleofection, the viability of cells decreased from 96.70% to 38.83% ± 1.76% and 75.83% ± 4.14% for the nucleofected cells with and without the RNP and ssODN, respectively (Fig. 1c). The lowest viability of the nucleofected cells with the RNP and ssODN was observed on day 4 after nucleofection at 35.13% ± 9.73% (Fig. 1c). The viability of the nucleofected cells with the RNP and ssODN was significantly lower than those of the untransfected cells and the nucleofected cells without the RNP and ssODN throughout the 10-day culture period (Fig. 1c). When the transfected cells recovered from being nucleofected, the genomic DNA was isolated for PCR. DNA sequencing data revealed that the HbE mutation (**A**AG) identified in the untransfected cells and the nucleofected cells without the RNP and ssODN was not observed in the nucleofected cells with the RNP and ssODN (n = 3) (Fig. 1d), indicating the highly efficient genetic correction of HbE mutation in the SiBBE cells. The HbE-corrected SiBBE pool was then subjected to clonal isolation. Five subclones, designated as A8, B3, B9, D10 and G5, were obtained from the clonal isolation and were maintained in the expansion medium for further validation.

### Clonal validation by multiplex PCR analysis of HbE mutation and off-target analysis

After clonal expansion, we performed multiplex PCR analysis for HbE mutation in five subclones according to the previous publications^15,18^. The parental SiBBE cells had a product size of 529 bp representing the HbE point mutation, while this band disappeared in all five subclones (Fig. 1e). When we performed DNA sequencing analyses of these five subclones, we determined that four of them were corrected from heterozygous HbE (AAG) to the wild-type allele (GAG). However, one subclone, A8, had a heterozygous 3-bp deletion around the CRISPR cut site (Supplementary Fig. 1). Of these, we picked three clones (B3, B9 and D10) for off-target analysis. DNA sequencing data revealed that there were no indel mutations in all the predicted exonic off-target sites: *HBD*, *RNF213*, *EDEM2* and *NADHA* (*COA*) (Fig. 1f).

### Erythroid differentiation of the HbE-corrected SiBBE cells

**Figure 2:**
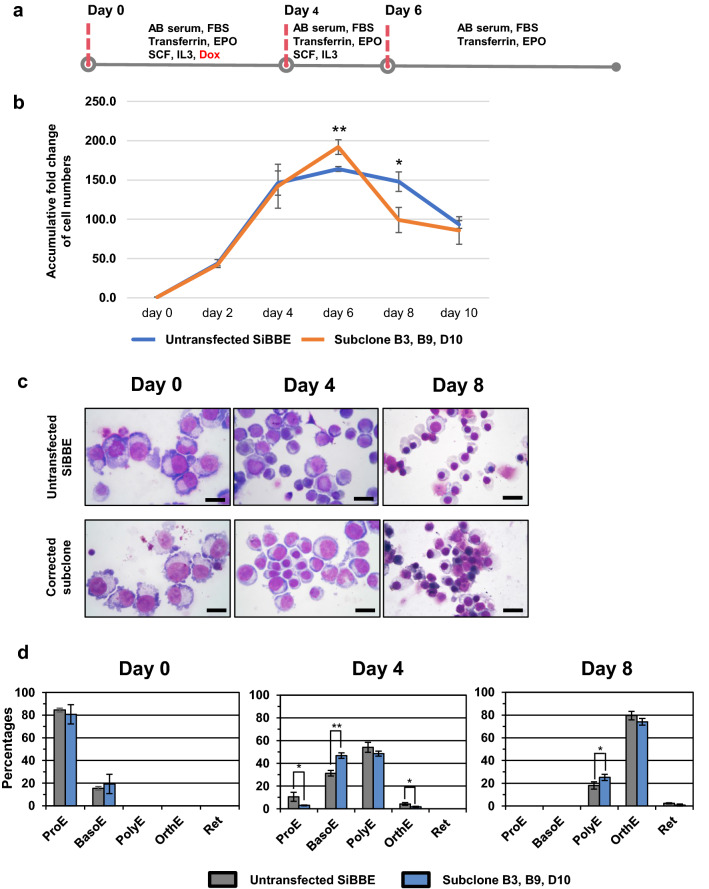
Erythroid differentiation of the HbE-corrected SiBBE cells. (**a**) Schematic diagram of the erythroid differentiation protocol used in this study. (**b**) Accumulative fold expansion during erythroid differentiation of the HbE-corrected SiBBE subclones (B3, B9 and D10) and the untransfected SiBBE cells. (**c**) The differentiated HbE-corrected SiBBE subclone and untransfected SiBBE cells on days 0, 4 and 8 stained with Leishman reagent and analysed by a light microscope; representative images of three independent cultures (scale bar = 20 µm). (**d**) Percentage of erythroid cell types on days 0, 4 and 8 during erythroid differentiation of the HbE-corrected SiBBE subclones and the untransfected SiBBE cells (mean ± SD, n = 3). ProE = proerythroblast; BasoE = basophilic erythroblast; PolyE = polychromatic erythroblast; OrthE = orthochromatic erythroblast; Ret = reticulocyte. *p value < 0.05, **p value < 0.01, two-tailed t-test.

To determine whether β-globin production was restored in the edited cells, the three HbE-corrected SiBBE subclones (B3, B9 and D10) were differentiated toward erythroid cells (Fig. 2a). The untransfected SiBBE pool was also used as a control. The cells were transferred to the primary medium of the erythroid culture system^19^ for 6 days, with doxycycline for days 0–4, followed by a tertiary medium of the same system thereafter. The numbers of the HbE-corrected cells were significantly higher than those of the untransfected SiBBE cells on day 6 but lower than those of the untransfected SiBBE cells on day 8. However, there was no difference in the numbers of cells between the HbE-corrected and untransfected SiBBE cells at the end of the culture (day 10) (Fig. 2b).

Morphological characterisation demonstrated that both the HbE-corrected and untransfected SiBBE cells followed similar differentiation stages during expansion, with the majority of cells (more than 80%) being proerythroblasts and the remainder basophilic erythroblasts (Fig. 2c and d). Further erythroid differentiation of the HbE-corrected SiBBE cells showed a slight difference in cell types compared to the untransfected control. The predominant erythroid populations of the HbE-corrected SiBBE cells were basophilic erythroblasts on day 4, with significantly higher basophilic erythroblasts (*p* value = 0.0015; n = 3), as well as lower proerythroblasts (*p* value = 0.1238; n = 3) and orthochromatic erythroblasts (*p* value = 0.0242; n = 3) than those of the untransfected SiBBE cells. On day 8, the HbE-corrected SiBBE cultures also had significantly higher polychromatic erythroblasts (*p* value = 0.0451; n = 3) than those of the untransfected SiBBE cells. However, there was no significant difference (*p* value = 0.058, n = 3) in the numbers of enucleated cells between the corrected SiBBE and untransfected SiBBE cells (Fig. 2d).

**Figure 3:**
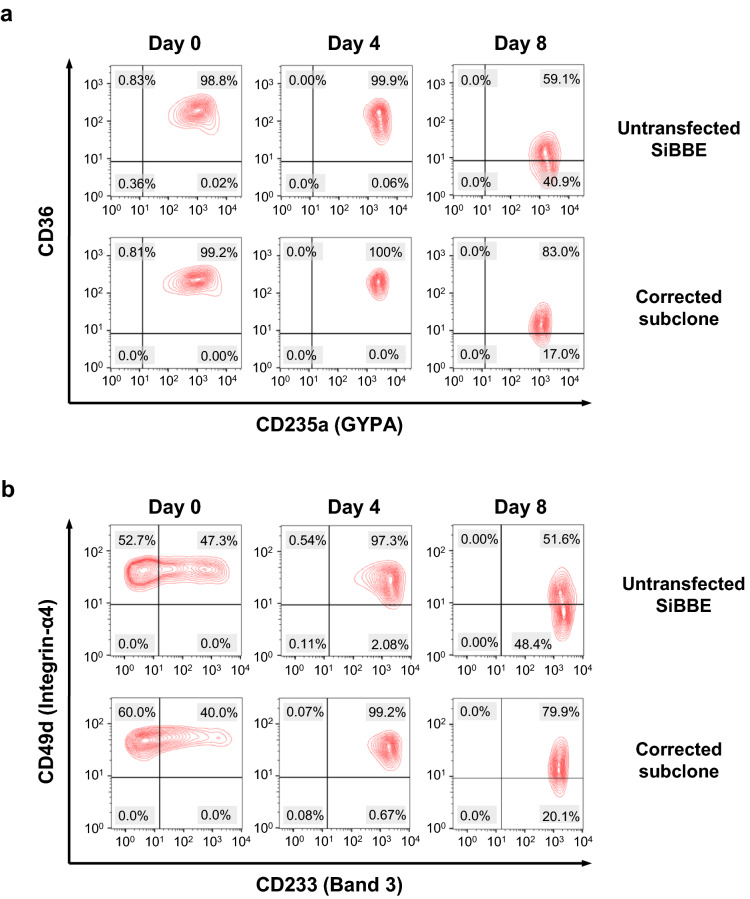
Erythroid marker expression of the HbE-corrected SiBBE cells. (**a**) Expression of CD235a (GYPA) and CD36 on days 0, 4 and 8 of erythroid differentiation of the HbE-corrected SiBBE subclone and the untransfected SiBBE cells as analysed by flow cytometry; representative plots of three independent cultures. (**b**) Expression of CD233 (Band3) and CD49d (integrin α4) on days 0, 4 and 8 of erythroid differentiation of the HbE-corrected SiBBE subclone and the untransfected SiBBE cells as analysed by flow cytometry; representative plots of three independent cultures. Figures were generated using the FlowJo v10 software (BD Biosciences, https://www.flowjo.com/solutions/flowjo/downloads).

Flow cytometric analysis was performed with antibodies against the key RBC membrane proteins (GYPA, CD36, α4-integrin and Band3). Similar to the expression pattern in other immortalised erythroid cells and cultured erythroblasts^20,21^, the expression of GYPA and Band3 in the HbE-corrected SiBBE cells increased following the maturation of erythroid cells. In contrast, the expression of CD36 and α4-integrin decreased following the maturation of erythroid cells (Fig. 3a and b). There was no difference in differentiation patterns between the HbE-corrected SiBBE cells and the control cells on day 0 and day 4 of differentiation. On day 8, the HbE-corrected cells were 85.60 ± 2.65% GYPA^+^ CD36^+^ with 14.40 ± 2.65% GYPA^+^ CD36^−^, which was significantly different from 60.53 ± 3.20% GYPA^+^ CD36^+^ (*p* value = 0.0004, n = 3) with 39.47 ± 3.20% GYPA^+^ CD36^−^ (*p* value = 0.0004, n = 3) in the untransfected SiBBE cells. Similar to the GYPA and CD36 expression, the HbE-corrected cells were 83.07 ± 2.94% CD233^+^ CD49d^+^ with 16.93 ± 2.94% CD233^+^ CD49d^−^, which was significantly different from 55.67 ± 3.72% CD233^+^ CD49d^+^ (*p* value = 0.0006, n = 3) with 44.33 ± 3.72% CD233^+^ CD49d^−^ (*p* value = 0.0006, n = 3) in the untransfected SiBBE cells. These results indicate a slight delay in the differentiation of the HbE-corrected SiBBE cells. Unfortunately, the viability of the HbE-corrected SiBBE cells dropped below 50% on day 10, therefore, the cells were not suitable for morphological analysis by Leishman’s eosin methylene blue staining or flow cytometry.

### Haemoglobin expression in the HbE-corrected SiBBE cells

**Figure 4:**
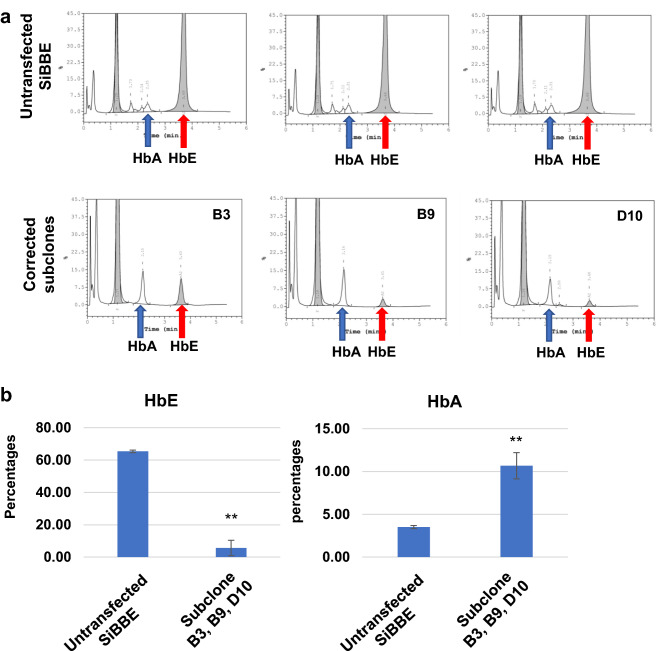
Haemoglobin expression of the HbE-corrected SiBBE cells. (**a**) HPLC traces showing HbA and HbE levels in the three HbE-corrected subclones (B3, B9 and D10) (lower panel) and untransfected SiBBE cells (upper panel). (**b**) Percentage of HbA and HbE expression in the HbE-corrected SiBBE subclones and the untransfected SiBBE cells **p value < 0.01, two-tailed t-test.

We further evaluated the haemoglobin (Hb) proteins in the erythroid cells on day 8 of differentiation from both the untransfected SiBBE cells and the HbE-corrected subclones by HPLC analysis. The untransfected SiBBE cells showed 65.3 ± 0.8% HbE, 3.5% ± 0.2% HbA and the rest were HbF. As expected from the HbE correction, HbE significantly decreased in the HbE-corrected cells (5.6 ± 4.7%, *p* value = 0.00003, n = 3) and HbA significantly increased in HbE corrected cells (10.7 ± 1.5%, *p* value = 0.001, n = 3) (Fig. 4a and b).

## Discussion

HbE/β-thalassaemia is a common subtype of β-thalassemia, which causes severe clinical syndrome in the patients^4^. Most patients with HbE/β-thalassaemia suffer from marked anaemia, jaundice, hepatosplenomegaly, growth retardation, and thalassaemic facies; and require regular blood transfusion from early life with resultant consequences of iron overload and allo-immunisation^6^. Blood shortage is also a major concern in several countries, especially in low- to middle-income countries where the prevalence of β-thalassaemia is high^22^. Some medications have been used to increase foetal haemoglobin expression; however, the effectiveness of such treatments varies among individuals^6,23–27^. Bone marrow transplantation is the only curative and definitive treatment but is limited to only patients who have compatible stem cell donors^28,29^. Therefore, genetic correction of the β-globin mutations in the patient stem cells followed by autologous transplantation has become an ideal solution as a curative treatment of β-thalassaemia^30^.

Patient-derived iPSCs offer promise for cell-based therapy. However, in the case of genetic diseases, genetic correction of mutations is required prior to directed differentiation into disease-affected cell types. In the past decade, several studies reported the genetic correction of β-globin mutations in thalassaemia patient-derived iPSCs using different gene editing technologies. Some studies showed the restoration of *HBB* transcript by RT-PCR or quantitative RT-PCR analysis in either the corrected iPSC-derived colony-forming unit erythroid (CFU-E) or the differentiated erythroid cells from culture^9,12–14^. Other studies showed the expression of the β-globin protein in the corrected-iPSC-derived erythroblasts by Western blot analysis^8,10,11,15^. It is worth noting that most of the erythroblasts were immature and only expressed epsilon- and gamma-globin rather than adult globin protein. This is due to the lack of important transcription factors, KLF1 and BCL-11A, responsible for globin switching in erythroid cells differentiated from iPSCs^15,31^. In addition, the number of differentiated erythroid cells is limited, making the HPLC analysis of globin typing very challenging. Recently, we generated an immortalised erythroid cell line from stem cells of a patient with HbE/β-thalassaemia, SiBBE^17^. Unlike the findings on iPSCs, the erythroid cells differentiated from this cell line express KLF1 and BCL-11A, thereby exhibiting a similar globin profile to those of the patient’s red blood cells. Thus, the SiBBE cell line represents a suitable model for studying the genetic correction of β-globin mutations.

In this study, we first used the plasmid-based CRISPR/Cas9 system with the ssODN donor template, which was previously used for correcting HbE mutation in the iPSCs derived from the HbE/β-thalassaemia patient^15,18^, for correcting the HbE mutation in the SiBBE cell line. However, we did not obtain any HbE-edited clones, and the percentage of viability of the transfected SiBBE cells after nucleofection was very low. Previous studies demonstrated that using Cas9 and sgRNA plasmid-based transfection could result in direct toxicity to the cells, which may cause cell death^32,33^. Therefore, we modified the system to the RNP complex consisting of Cas9 nuclease and the in vitro transcribed (IVT) sgRNA, which has been shown to improve knockout efficiency^32,34^. In addition, we also modified the ssODN donor template with phosphorothioate to increase the stability of the ssODN and enhance the knock-in efficiency during DNA repair^35^. Using this approach, we obtained very high HDR-mediated correction according to the ICE analysis. When we isolated five subclones from the pool, DNA sequencing revealed that 4 subclones had HDR-mediated correction of the HbE allele, while one subclone had an NHEJ-mediated indel mutation around the CRISPR cut site on the allele that previously had the HbE mutation. Of these five subclones, three were selected for further analysis. Notably, there were no indel mutations in all the potential off-target sites, including *HBD* gene, which has similar homology to the *HBB* gene. In contrast, the previous study in the HbE/β-thalassaemia**-**iPSCs employing the plasmid-based CRISPR/Cas9 system for correcting HbE mutation identified a point mutation in two of the three iPSC clones screened, although these mutations did not affect the haematopoietic differentiation. This result supports the notion that using the RNP complex reduces off-target mutations in both primary and stem cells^32,34^. The disadvantage of this method may be that the percentage of viability of the SiBBE cells after nucleofection was still low as a result of a combination of nucleofection and the delivery of the RNP complex and ssODN into the cells. However, since the SiBBE cells have an unlimited self-renewal property, cell numbers could simply be restored after cell recovery from nucleofection (as shown in Fig. 1b). Further optimisation of nucleofection parameters could be done to improve cell viability after transfection in the future.

Our previous studies demonstrated the advantages of the immortalised erythroid cell line over other models such as CD34^+^ haematopoietic stem/progenitor cells and iPSCs in terms of the proliferative ability of the erythroid cells upon differentiation^17,36^ and the capability for genome editing^37^. We applied the same erythroid culture system to generate a sufficient amount of differentiated erythroid cells and determine if the beta-globin (HbA) protein was restored. In contrast to the findings found in the iPSCs^15^, the expression of HbE diminished in the corrected SiBBE cells; the remaining 5% of HbE could be a trace from HbA2 as both HbE and HbA2 share the exact same location on the HPLC trace. Moreover, we showed for the first time a significant increase in HbA protein production in the HbE-corrected erythroid cells. However, the amount of HbA in the HbE-corrected SiBBE cells did not increase to the normal level. Although a β-globin mutation on the other allele remains, it is not common for the cells to produce such a high HbF level instead of HbA. One possible cause is a large deletion at the beta-globin gene on the other allele after the genetic correction; therefore, we perform sequencing to show that there was no large deletion at the beta-globin gene and its promotor sequence. The heterozygous mutations previously reported in the SiBBE cells were still detected after HbE correction, which suggests that both alleles of the beta-globin gene were still present and that there was likely to be no large deletion that would eliminate the non-corrected allele of this locus after the HbE correction (supplementary Fig. 2)^17^. In addition, the numbers of both HbE-corrected and control SiBBE cells decreased after day 6 in the culture when most of the cells were polychromatic erythroblasts. This finding is in line with those observed in the erythroid cells differentiated from β-thalassaemia patient HSCs due to ineffective erythropoiesis^38^. It appears likely that the effect of ineffective erythropoiesis remains in the HbE-corrected SiBBE cells; therefore, further experiments are needed to identify other possible causes, such as aberrant expression of important transcription factors that regulate β-globin expression.

The unlimited proliferation ability of the gene-edited immortalised SiBBE cell line offers great opportunity to generate clinically relevant red blood cell products for treating thalassaemia patients without allo-immunisation consequences. Our approach is also applicable to other genetic blood diseases such as sickle cell anaemia. In addition, the risk from using viral oncogenes to immortalise patients’ erythroblasts is not a concern as their expression is lost upon differentiation. Furthermore, red blood cells do not have nuclei; therefore, the risk of tumour transformation is diminished^36^.

In conclusion, we utilised CRISPR/Cas9 genome editing technology to efficiently correct the HbE mutation in the immortalised erythroid cell line, SiBBE, and demonstrated a proof of principle that the HbE production diminished once the mutation was corrected. Additionally, the HbE-corrected erythroid cells produced significantly increased adult haemoglobin. Altogether, the patient-derived immortalised erythroid cell line represents a valuable study model for genetic correction and provides a sustainable source of red blood cells for cell-based therapy in thalassaemia.

## Methods

### Cell culture

Generation of the SiBBE cell line was performed in accordance with the Declaration of Helsinki and after approval by the local research ethics committees (The Siriraj Institutional Review Board, SIRB; COA no. SI159/2018)^17^. Written informed consent was obtained from a donor who had been diagnosed as HbE/β-thalassaemia. The immortalised erythroid cell line, SiBBE, derived from a HbE/β-thalassaemia patient, was maintained in the culture medium: StemSpan™ SFEM medium (STEMCELL Technologies) supplemented with 3 U/ml erythropoietin (EPO) (Roche), 10^−6^ M dexamethasone (Sigma-Aldrich), 50 ng/ml stem cell factor (SCF) (R&D Systems) and 1 µg/ml doxycycline (Takara Bio). The cells were counted using trypan blue staining, maintained at a density of 2–3 × 10^5^ cells/ml, and cultured at 37 °C, 5% CO_2_ with a total medium change performed every other day. For erythroid differentiation, the cells were transferred to primary medium^19^, which was a basic medium (Iscove’s medium (Biochrom) containing 3% (v/v) human AB serum (Sigma-Aldrich), 2% fetal calf serum (Hyclone, Fisher Scientific), 3 U/ml EPO (Roche), 200 μg/ml transferrin (R&D Systems) and 1 U/ml penicillin/streptomycin (Sigma-Aldrich)) supplemented with 10 ng/ml SCF (R&D Systems) and 1 ng/ml IL-3 (R&D Systems), and maintained for 6 days. On days 0–4, 1 µg/ml of doxycycline was added to the culture medium. After day 6, the cells were transferred to and maintained in a tertiary medium, which was the basic medium supplemented with 500 μg/ml transferrin^19^. The cells were counted using trypan blue staining, maintained at a density of 2–3 × 10^5^ cells/ml for the first 6 days and at a density of 1–2 × 10^6^ cells/ml thereafter, at 37 °C, 5% CO_2_ with total medium change performed every other day.

### Genetic correction of HbE using CRISPR/Cas9 ribonucleoprotein complex (RNP) system

Genetic correction of the HbE mutation (**A**AG) was performed using the gRNA sequence previously described in our published work (3, 4) and the 90 base-pair long phosphorothioate-modified single-stranded oligonucleotide (ssODN) containing the left homology arm (LHA), the correct nucleotide (**G**AG) and the right homology arm (Supplementary file 1). The single guide RNA (sgRNA) was synthesized using the GeneArt™ Precision gRNA Synthesis Kit (Invitrogen). For the RNP complex formation, 90 µg/ml sgRNA and 150 µg/ml Alt.R® S.p. Cas9 nuclease V3 (Integrated DNA technologies) (at a 3:1 molar ratio) were co-incubated for 10 min at room temperature. Briefly, 2 × 10^5^ SiBBE cells resuspended in 20 µl P3 Primary Cell Solution were nucleofected with the RNP complex and the ssODN donor template in the Nucleocuvette™ Strip using the Amaxa 4D-nucleofector system with the program FF-120 (Lonza). The cells were quickly transferred to a well of 12-well plate containing the culture medium. Nine days post-nucleofection, the genomic DNA of the transfected SiBBE pool was isolated for polymerase chain reaction (PCR) using DreamTaq Green PCR master mix (Thermo Fisher Scientific) and the primers listed in Supplementary file 1. The cycle parameters were initial denaturation at 95 °C for 3 min, denaturation at 95 °C for 30 s, annealing at 68 °C for 45 s, extension at 72 °C for 1 min, and final extension at 72 °C for 5 min. DNA sequencing data of the amplicon was compared to the wild-type sequence using Inference of CRISPR Edits (ICE) analysis.

(https://www.synthego.com/products/bioinformatics/crispr-analysis).

### Clonal isolation by limiting dilution

Clonal isolation of the transfected SiBBE pool was performed on day 9 post-nucleofection. The transfected cells were diluted to a concentration of 10 cells/ml. An aliquot of 100 µl of the cell suspension was seeded onto a well of 96-well plate. Only wells that contained 1 cell were expanded for clonal validation by multiplex PCR analysis and further experimentation.

### Clonal validation

Multiplex PCR analysis of the edited clones was performed using the primers listed in Supplementary file 1. The HbE negative clones were selected for further validation by Sanger sequencing. In addition, the four predicted exonic off-target sites were PCR amplified using the primers listed in Supplementary file 1. Cycle parameters were initial denaturation at 98 °C for 30 s, denaturation at 98 °C for 10 s, annealing at 68 °C for 30 s, extension at 72 °C for 30 s, and final extension at 72 °C for 2 min. The amplicon was analysed by Sanger sequencing. The DNA sequencing data were compared with the wild-type sequence using ICE analysis. Additionally, possible large deletions in the edited clones were examined by PCR of the genomic sequence of the HBB gene and Sanger sequencing of the amplicons. Primers were also listed in Supplementary file 1.

### Flow cytometry

Cultured cells at selected time points were harvested and washed once in PBS-AG (PBS containing 1 mg/ml BSA and 2 mg/ml glucose). The cell pellet was then resuspended and incubated with 1:1 dilution of respective primary antibodies against CD233 or CD235a (IBGRL) for 60 min at 4 °C. The cells were washed once in PBS-AG and incubated with 1:500 dilution of the rat anti-mouse IgG1-APC (Biolegend) for 30 min at 4 °C in the dark, followed by washing as above. The cells were then co-stained with 1:10 dilution of the FITC-conjugated antibodies against CD36 and CD49d (Biolegend) for 30 min at 4 °C in the dark. Flow cytometric data were acquired using FACSCalibur (BD Biosciences) and analysed using the FlowJo v10 software (BD Biosciences).

### Cytospin

An aliquot of 10^4^ to 10^5^ cells was diluted with the culture medium to 200 µl before centrifugation in the Cytospin™ 4 cytocentrifuge (Thermo Scientific) at 1350 rpm for 5 min. Slides were allowed to air dry before being fixed in methanol for 10–20 min. They were then stained by soaking in Leishman’s eosin methylene blue solution for 3 min, followed by 7 min in diluted Leishman’s eosin methylene blue solution (diluted 1:1). The stained slides were examined using a light microscope (Carl Zeiss AG) with a digital camera mounted.

## Supplementary Information

Supplementary Information.

## Data Availability

Any data or material that support the findings of this study can be made available by the corresponding author upon request.
