# Supplementary material for: Genetic correction of haemoglobin E in an immortalised haemoglobin E/beta-thalassaemia cell line using the CRISPR/Cas9 system
Source: Sci Rep. 2022 Sep 16;12:15551. doi: 10.1038/s41598-022-19934-7 (PMC9481540; doi:10.1038/s41598-022-19934-7)
Supplement: Supplementary file 1 — Supplementary Information. [file 41598_2022_19934_MOESM1_ESM.docx]

**Genetic correction of haemoglobin E in an immortalised haemoglobin E/beta-thalassemia cell line using the CRISPR/Cas9 system**

Kongtana Trakarnsanga^1,^, Nontaphat Thongsin^2,3^, Chanatip Metheetrairut^1^, Chartsiam Tipgomut^1^, Saiphon Poldee^1^, Methichit Wattanapanitch^2*^

1 Department of Biochemistry, Faculty of Medicine Siriraj Hospital, Mahidol University, Bangkok, Thailand; kongtana.tra@mahidol.ac.th (K.T); chanatip.met@mahidol.ac.th (C.M.); chartsiam.tip@mahidol.ac.th (C.T.); sayphon.pho@mahidol.ac.th (S.P.)

2 Siriraj Center for Regenerative Medicine, Research Department, Faculty of Medicine Siriraj Hospital, Mahidol University, Bangkok, Thailand; nontaphat.tho@student.mahidol.edu (N.T.); methi-chit.wat@mahidol.ac.th (M.W.)

3 Department of Immunology, Faculty of Medicine Siriraj Hospital, Mahidol University, Bangkok, Thailand

* Correspondence: methichit.wat@mahidol.ac.th; Tel.: (+66 876718012)

**
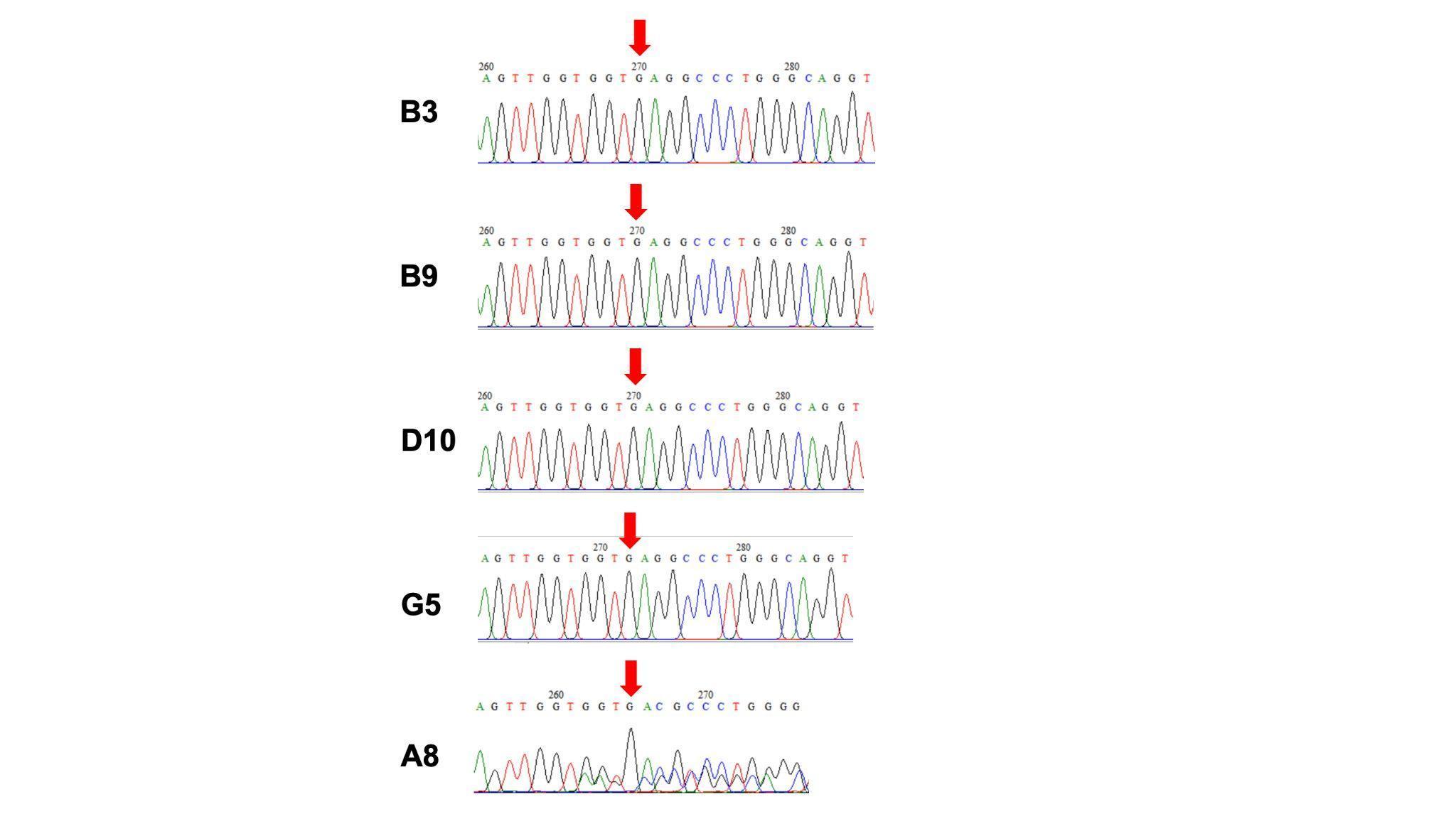
**

**Supplementary figure 1** Beta-globin gene was PCR amplified from genomic DNA of 5 corrected SiBBE subclones (B3, B9, D10, G5 and A8) and sequenced by standard Sanger sequencing. Heterozygous allele at *HBB*: c.79G>A in exon 1, creating haemoglobin E (HbE) allele, was not detected.

**A.**

**
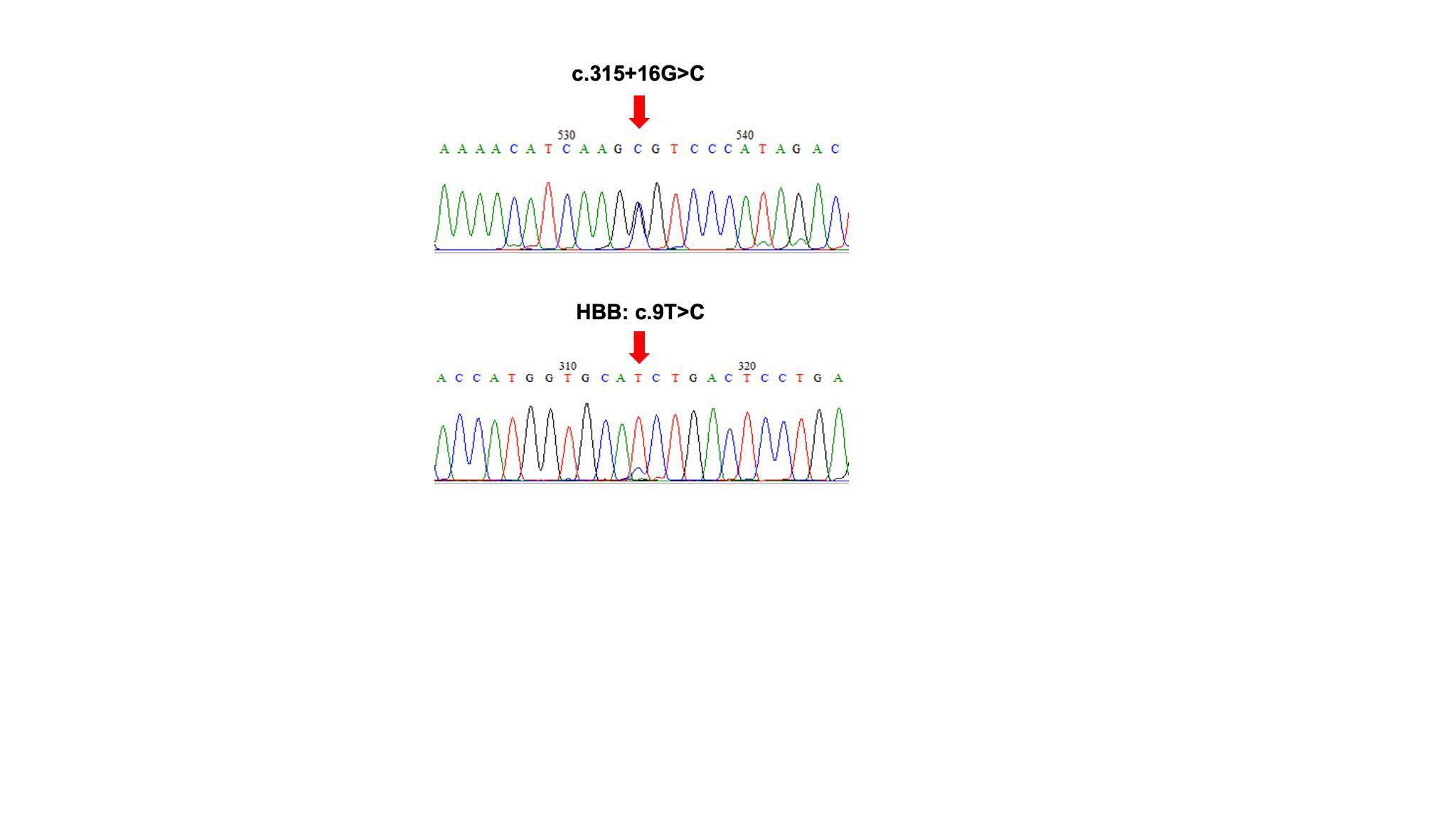
**

**B.**

**
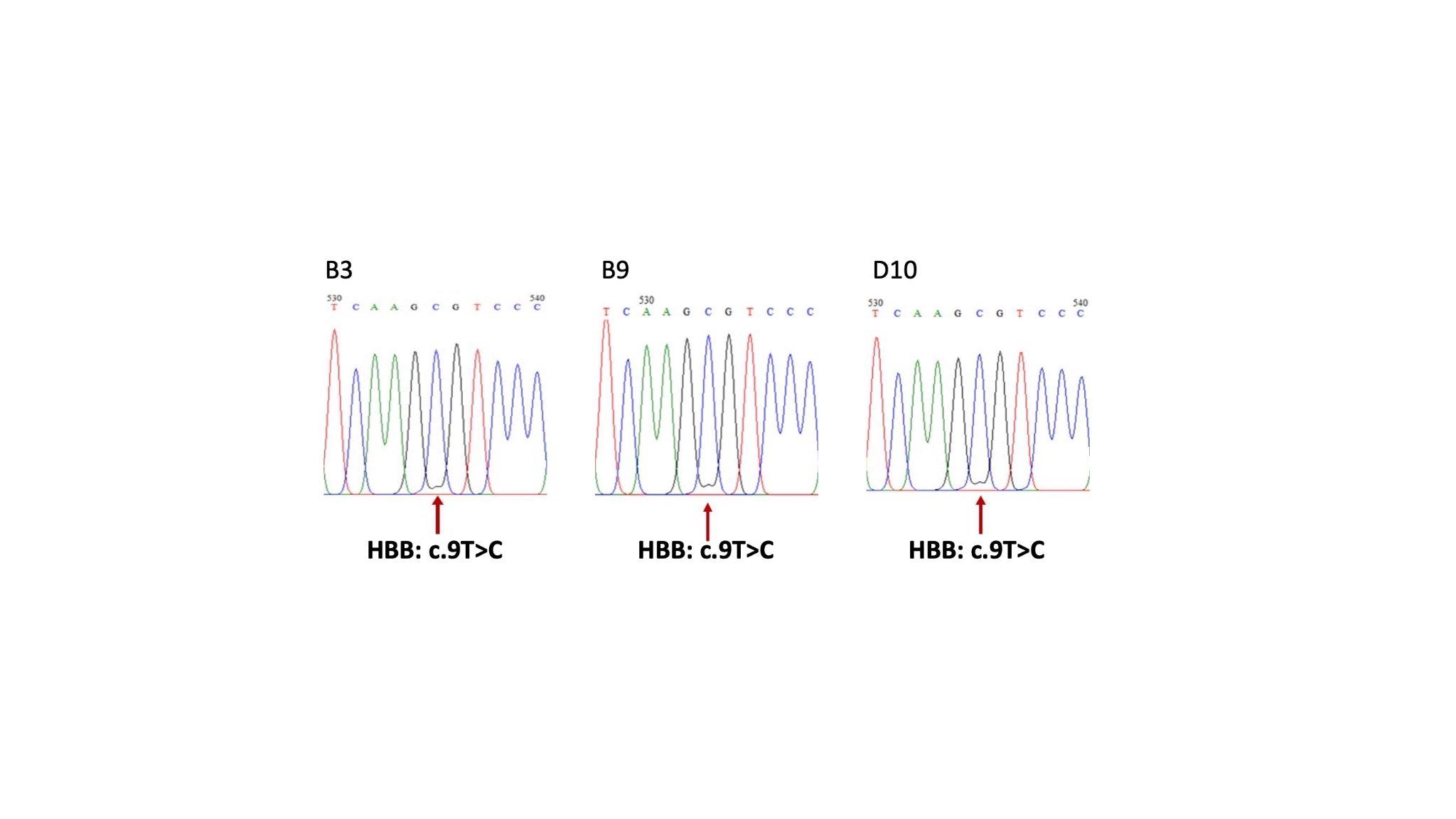
**

**Supplementary figure 2** Beta globin gene and its promotor region were amplified from genomic DNA of the corrected SiBBE pool and sequenced by standard Sanger sequencing. (A) Heterozygous mutations at *HBB*: c.9T>C, c.135+16G>C previously reported in the SIBBE cells were detected in the pooled corrected SiBBE cells. (B) Heterozygous mutations at *HBB*: c.9T>C were detected in the corrected SiBBE subclones.

**
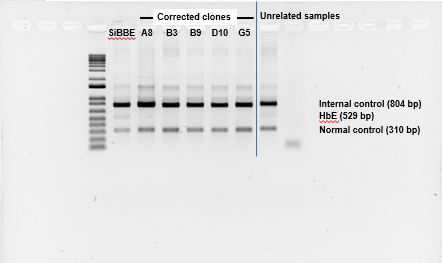
**

**Supplementary figure 3 Original gel image of the image in Figure 1e**

**Supplementary file 1.**

**gRNA sequence targeting HbE mutation**

5’-TGGATGAAGTTGGTGGTA-3’

**Oligo and primer sequences**

**ssODN sequence** (* represents phosphorothioate modification to increase DNA stability)

G*T*T*ACTGCCCTGTGGGGCAAGGTGAACGTGGATGAAGTTGGTGGTGAGGCCCTGGGCAGGTTGGTATCAAGGTTACAAGACAGGTTT*A*A*G

**Primers for DNA sequencing**

**HBB forward:** TCCAACTCCTAAGCCAGTGC

**HBB reverse:** CGATCCTGAGACTTCCACACTG

**Primers for multiplex PCR analysis of HbE**

**HbE-Fc:** TCCAACTCCTAAGCCAGTGC

**HbE-Rn:** CCTGCCCAGGGCCTC

**HbE-Fm:** CGTGGATGAAGTTGGTGGTA

**HbE-Rc:** CGATCCTGAGACTTCCACACTG

**Primers for off-target analysis**

| **Off-target** | **Sequences (5’ 🡪 3’)** |
| --- | --- |
| **HBD** | Forward: AACCAACCTGCTCACTGGAG |
|  | Reverse: AGCCTTCACCTTAGGGTTGC |
| **RNF213** | Forward: TGGAAGCAGGTGGACAGTTC |
|  | Reverse: TGTGTAGGTTACCCAAGGCAC |
| **EDEM2** | Forward: AACCGCATGGAGTCGTTCTT |
|  | Reverse: ATGGCCCCGAACTAACAGTG |
| **HADHA (COA)** | Forward: GAGTTGACCCGAAGAAGCTG |
|  | Reverse: CAGGCTGGCCTCACACTTCT |

**Primers for identifying possible large deletion in HBB gene**^17^

**FW1:** CTTACCAAGCTGTGATTCCAAA

**RV1:** CTTCATCCACGTTCACCTTG

**FW2:** GCTTCTGACACAACTGTGTTC

**RV3:** GCCCTGAAAGAAAGAGATTAGG

**FW4:** CACATATTGACCAAATCAGGGT

**RV4:** GGCAGAATCCAGATGCTCAA
